# Screening for novel risk factors related to high myopia using machine learning

**DOI:** 10.1186/s12886-022-02627-0

**Published:** 2022-10-13

**Authors:** Ruiheng Zhang, Li Dong, Qiong Yang, Wenda Zhou, Haotian Wu, Yifan Li, Heyan Li, Wenbin Wei

**Affiliations:** Beijing Key Laboratory of Intraocular Tumor Diagnosis and Treatment, Beijing Ophthalmology & Visual Sciences Key Lab, Medical Artificial Intelligence Research and Verification Key Laboratory of the Ministry of Industry and Information Technology, Beijing Tongren Hospital, Beijing Tongren Eye Center, Capital Medical University, Beijing, China

**Keywords:** Machine learning, Myopia, High myopia, Vitamin A

## Abstract

**Background:**

High myopia-related complications have become a major cause of irreversible vision loss. Evaluating the association between potential factors and high myopia can provide insights into pathophysiologic mechanisms and further intervention targets for myopia progression.

**Method:**

Participants aged 12–25 years from National Health and Nutrition Examination Survey 2001–2006 were selected for the analysis. Myopia was defined as spherical equivalent (sum of spherical error and half of the cylindrical error) of any eyes ≤-0.5 diopters. High myopia was defined as the spherical equivalent of any eye ≤ − 5.00 diopters. Essential variables were selected by Random Forest algorithm and verified by multivariable logistic regression.

**Results:**

A total of 7,033 participants and 74 potential factors, including demographic (4 factors), physical examination (6 factors), nutritional and serological (45 factors), immunological (9 variables), and past medical history factors (10 factors), were included into the analysis. Random Forest algorithm found that several anthropometric, nutritional, and serological factors were associated with high myopia. Combined with multivariable logistic regression, high levels of serum vitamin A was significantly associated with an increased prevalence of high myopia (adjusted odd ratio = 1.46 for 1 µmol/L increment, 95% confidence interval [CI] 1.01–2.10). Furthermore, we found that neither C-reactive protein nor asthma increased the risk and severity of myopia.

**Conclusion:**

High levels of serum vitamin A was seemingly associated with an increased prevalence of high myopia. This borderline significant association should be interpreted with caution because the potential increased type I error after the multiple testing. It still needs further investigation regarding the mechanism underlying this association. Neither C-reactive protein nor asthma increased the risk and severity of myopia.

**Supplementary Information:**

The online version contains supplementary material available at 10.1186/s12886-022-02627-0.

**Summary Box**:

What was known before:

- It is estimated that 9.8% of the global population will have high myopia in 2050.
- High myopia and related complications have become the primary cause of irreversible vision loss.

What this study adds:

- In the present study, we selected participants aged 12–25 and 74 factors from the US National Health and Nutrition Examination Survey 2001–2006, a multistage probability sample of the noninstitutionalized US population.
- After adjusting for age, sex, ethnicity, TV/computer usage, serum vitamin D level, and education attainment, every 1 µmol/L increment of serum vitamin A was seemingly associated with an increased prevalence of high myopia (odd ratio = 1.46, 95% confidence interval 1.01–2.10). This borderline significant association should be interpreted with caution because the potential increased type I error after the multiple testing.
- Neither C-reactive protein nor asthma was associated with myopia or high myopia.

## Introduction

It is estimated that 5.2% of the global population will have high myopia in 2020, and the number will increase to 9.8% in 2050 [1]. High myopia increases the risk of pathologic ocular changes such as myopic maculopathy, glaucoma, and retinal detachment, all of which can cause irreversible vision loss [2]. In the Beijing Eye Study, 43.6% of irreversible vision loss and blindness were caused by high myopia-related complications [3]. For now, high myopia-related complications have become the leading cause of irreversible vision loss and blindness globally, particularly in East Asia [4–7].

There are limited interventions that can delay or prevent high myopia-related complications [8]. Outdoor activity has been proven effective in preventing the onset of myopia. In He’s study, every additional 40 min of outdoor activity decreased the 3-year cumulative incidence rate by 9.1% [9]. Yet, a meta-analysis did not find a relationship between time outdoors and myopic progression [10]. Compared to single-vision spectacles, Orthokeratology or low dose of atropine may slow the progression of myopia. Orthokeratology benefits in slowing axial elongation (mean difference: -0.28 mm) during 2 years of follow-up [11]. Meanwhile, orthokeratology requires more office visits to ensure appropriate fit and monitor for corneal complications (such as bacterial keratitis), which limits the widespread use in myopia control [12]. Low dose of atropine exhibited a promising protection effect in reducing myopia progression, yet 51.3% and 13.2% of children received 0.01% atropine progressed by more than 0.50 D and 1.00 D after 12 months according to a recent trial in China [13, 14]. A thorough exploration of association between multi-dimensional factors and myopia could help to identify novel risk factors and potential intervention targets.

This study evaluated the association between a wide range of demographic, physical examination, nutritional and serological, immunological, past medical history exposures, and high myopia using the US National Health and Nutrition Examination Survey (NHANES), a nationally representative, cross-sectional biannual health survey. Such an approach can provide insight into potential intervention targets and pathophysiologic mechanisms of myopia progression.

## Method

### Data source and participants

The NHANES serial cross-sectional survey was conducted following the ethical standards of the declaration of Helsinki. The NHANES Institutional Review Board/NCHS Research Ethics Review Board approved the conduct of the NHANES serial cross-sectional survey, and documented consent was obtained from participants. Data analyzed in the present study was obtained from the NHANES 2001–2006 data files. Because all individually identifiable information has been removed from the NHANES dataset, additional ethical approval and consent to participate are exempted. We chose 2001 through 2006 for inclusion because of the consistent refraction examination. The NHANES, described in detail elsewhere [15], is a multistage probability sample of the noninstitutionalized US population and allows estimates representing the US population. A total of 31,509 participants attended the NHANES from 2001 to 2006. We excluded individuals aged > 25 (14,208 participants), without refraction examination results (9,105 participants), hyperopia (defined as refractive error > 0.5D in any eye, 887 participants), and without information regarding education attainment or TV/computer usage time (276 participants). Written informed consent and ethical approval were exempted because NHANES data is anonymous.

### Exposures and outcomes

All participants aged 12–25 were eligible to participate in the non-cycloplegic vision examination, including presenting distance visual acuity and objective refraction, which were examined using an autorefractor/keratometry (Nidek ARK-760 A, Nidek Co. Ltd., Tokyo, Japan). The mean of three consecutive measurements was recorded for each eye. Myopia was defined as spherical equivalent (sum of spherical error and half of cylindrical error) of any eye ≤-0.5 diopters (D). Then, high myopia was defined as the spherical equivalent of any eye ≤ − 5.00D.

There was a total of 74 factors (Supplementary Table 1), including demographic (4 factors), physical examination (6 factors), nutritional and serological (45 factors), immunological (9 variables), and past medical history factors (10 factors). The methodological details were described on the NHANES website (https://wwwn.cdc.gov/nchs/nhanes/continuousnhanes/). We utilized the metabolic equivalent of task score (METs) for physical activity measuring. METs were calculated as the sum: (a) walked or bicycled weekly; (b) tasks in or around home or yard every week; (c) muscle-strengthening activities every week; (d) play or exercise hard every week; e., the average level of physical activity daily. This physical activity time was transformed into hours per week multiplied by the suggested score. For the average level of physical activity every day, we assumed 40 h per week.

### Statistical analysis

Statistical analysis was performed in Stata version 15.0 (StataCorp LLC, College Station, TX, USA) and R version 4.3.0 (The R Foundation for Statistical Computing, www.R-project.org). T-test and chi-square were used to determine differences in cross-sectional characteristics between continuous and categorical data groups. We transferred the MET score into quartile data. A random forest model with 600 trees was used to identify the most important classified variables in the dataset (Randomforest package). Multiple imputations with median values impute missing clinical data in the derivation dataset. A Mean Decreased Accuracy score was used for the Random Forest model to select the most important variables. Multivariable logistic analysis was then used to evaluate the association between important variables with high myopia. We used a two-way scatter plot and component-plus-residual plot to demonstrate the association before and after adjusting for covariables. We also used a restrictive cubic spline to detect any non-linear association. All analyses were weighted to represent the US population and account for the intricate survey design. A 2-sided P < 0.05 was considered statistical significance.

## Results

### Characteristics of included participants

Seven thousand thirty-three participants aged 12 to 25 were included in this study (Table 1). Compared to non-myopia, participants with high myopia have significantly higher age and educational attainment and are more likely to be female. Additional cross-sectional comparisons of non-myopia, mild-to-moderate myopia, and high myopia participants reveal potential differences between groups on multiple variables, such as Triceps skinfold, cadmium, cotinine, HDL-cholesterol (Supplementary Table 2).

**Table 1:** Characteristics of included participants

	No myopia	Mild and moderate myopia	High myopia	P-trend
	n = 3205	n = 3459	n = 368	
Age	18.3 (0.1)	18.5 (0.1)	19.5 (0.3)	0.011
Gender (Female %)	47.0 (1.2)	50.6 (1.2)	62.5 (3.1)	< 0.001
Ethnicity %				0.02
Mexican American	11.9 (1.1)	12.4 (1.4)	6.9 (1.4)	
Other Hispanic	6.0 (1.4)	5.3 (0.8)	4.3 (1.4)	
Non-Hispanic White	63.4 (2.3)	61.3 (2.4)	66.8 (3.6)	
Non-Hispanic Black	13.7 (1.4)	14.3 (1.5)	11.2 (2.0)	
Other Race	5.0 (0.8)	6.7 (0.9)	10.9 (2.7)	
Education attainment %				< 0.001
< 9th Grade	27.6 (1.3)	26.5 (1.2)	15.5 (2.2)	
9-11th Grade	29.7 (1.2)	28.6 (1.1)	24.1 (2.7)	
High School Grade	17.5 (1.3)	16.6 (1.1)	19.8 (2.9)	
Some College or above	25.3 (1.5)	28.2 (1.4)	40.6 (3.7)	
TV and computer usage (hour/day)	2.97 (0.06)	3.06 (0.06)	3.11 (0.19)	0.21
MET scores				0.006
Low	19.3 (1.2)	19.4 (1.0)	14.0 (2.2)	
Low-middle	16.7 (0.9)	17.7 (1.0)	13.3 (1.8)	
Middle-high	27.8 (1.2)	29.4 (1.5)	40.7 (3.6)	
High	36.2 (1.3)	33.4 (1.4)	32.0 (3.2)	

Data were weighted estimates, and expressed as mean (standard error)

**Table 2:** Association between myopia and serum vitamin A

	Myopia			High Myopia		
	aOR	95% CI	P-value	aOR	95% CI	P-value
Age	1.01	0.98–1.04	0.53	1.02	0.94–1.10	0.65
Gender (Female vs. male)	1.18	1.02–1.36	0.027	1.71	1.25–2.33	0.001
Education attainment (per each category increment)	1.13	1.04–1.23	0.004	1.34	1.09–1.65	0.006
TV and computer usage > 2 h/day	1.07	0.91–1.26	0.42	1.07	0.72–1.58	0.73
MET scores (per 1 quarter increment)	0.9	0.82–0.98	0.015	0.85	0.70–1.04	0.12
Vitamin A (per 1 µmol/L increment)	1.01	0.83–1.24	0.88	1.46	1.01–2.10	0.045
Vitamin D (per 10 nmol/L increment)	0.96	0.91–1.02	0.16	0.90	0.83–0.98	0.012

Data were weighted estimates. aOR, adjusted odds ratio; 95% CI, 95% confidence interval. Multivariable logistic regression included all variables in the table

### Random forest algorithm identified potential associated factors with high myopia

**Fig. 1:**
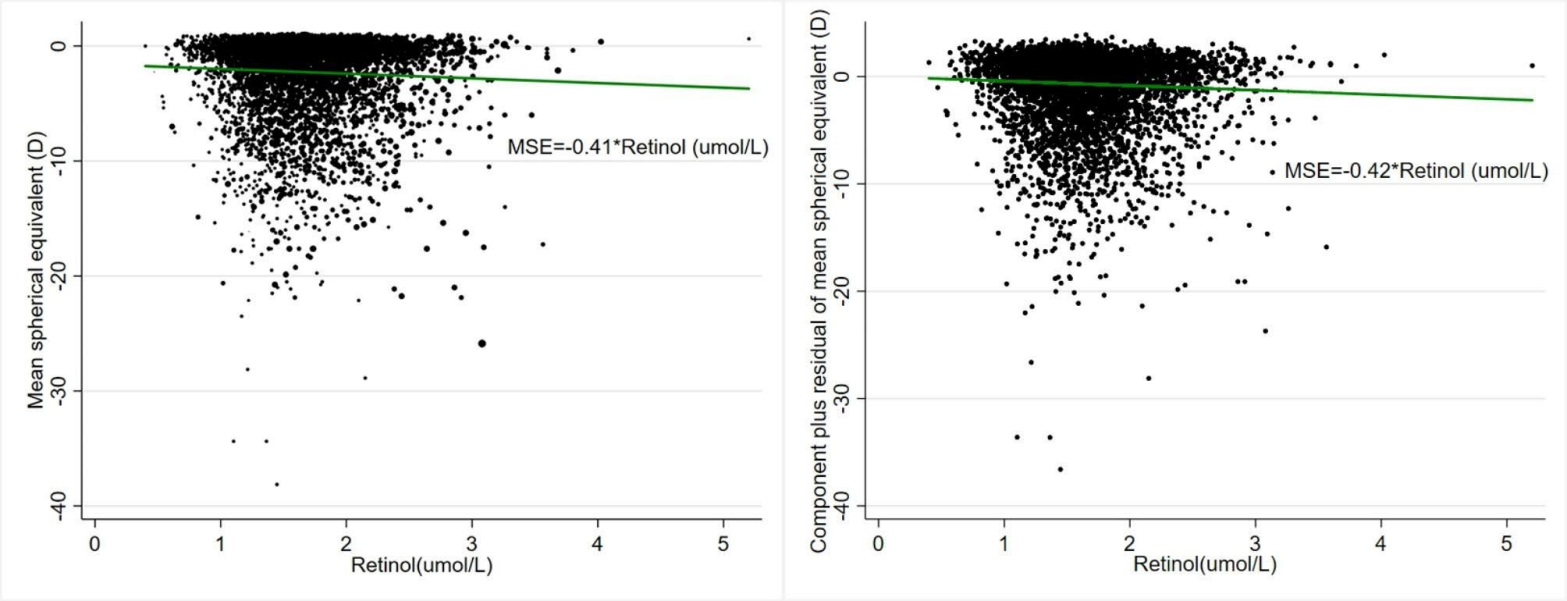
Mean decreased accuracy plot revealing the potential important variable

To avoid false-positive detection (type I error) and overcome the impact of collinearity and nonlinearity on the results of the logistics model, we applied Random Forest algorithm to select important potential factors [16]. The top 20 variables were selected by mean decreased accuracy plot (Fig. 1). Apart from traditional well-known risk factors of high myopia (such as age and education attainment), we found several anthropometric measurements (BMI, waist circumference, triceps skinfold, subscapular skinfold), nutritional and serological factors (such as vitamin A, vitamin D, alkaline phosphatase) have high importance in predicting high myopia.

Assuming the association between anthropometric measurements and high myopia resulted from reverse causation. We performed an in-depth exploration regarding other top 20 important factors. After adjusting for age, sex, ethnicity, TV/computer usage, serum vitamin D level, and education attainment, we identified that high levels of serum vitamin A was associated with high myopia (Supplementary Table 3).

**Fig. 2:**
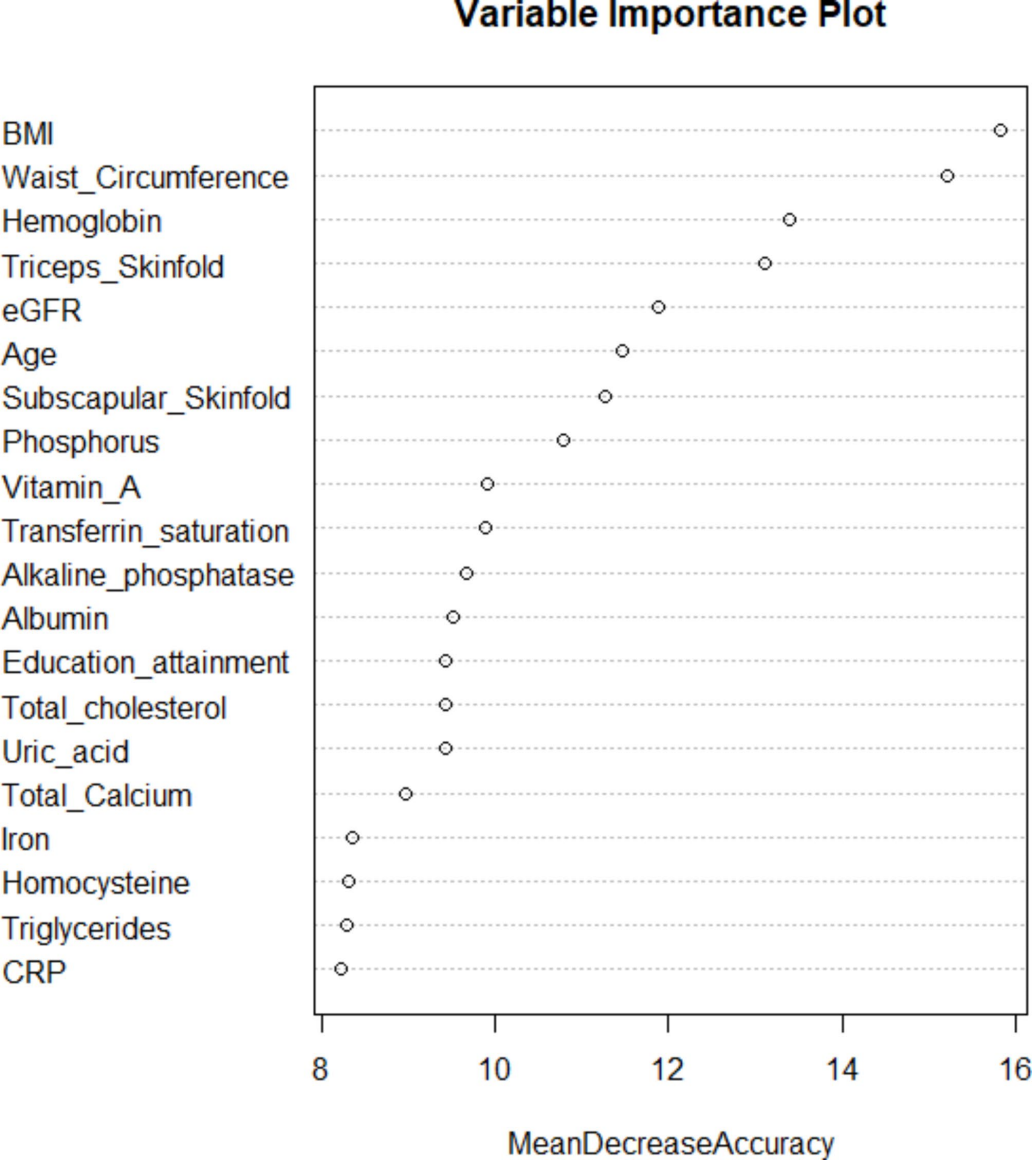
Scatter plot demonstration of the association between serum vitamin A and mean spherical equivalent (D), before (right) and after (left) adjusted for covariables. mean spherical equivalent is calculated by the mean of left and right eyes’ spherical equivalent

**Fig. 3:**
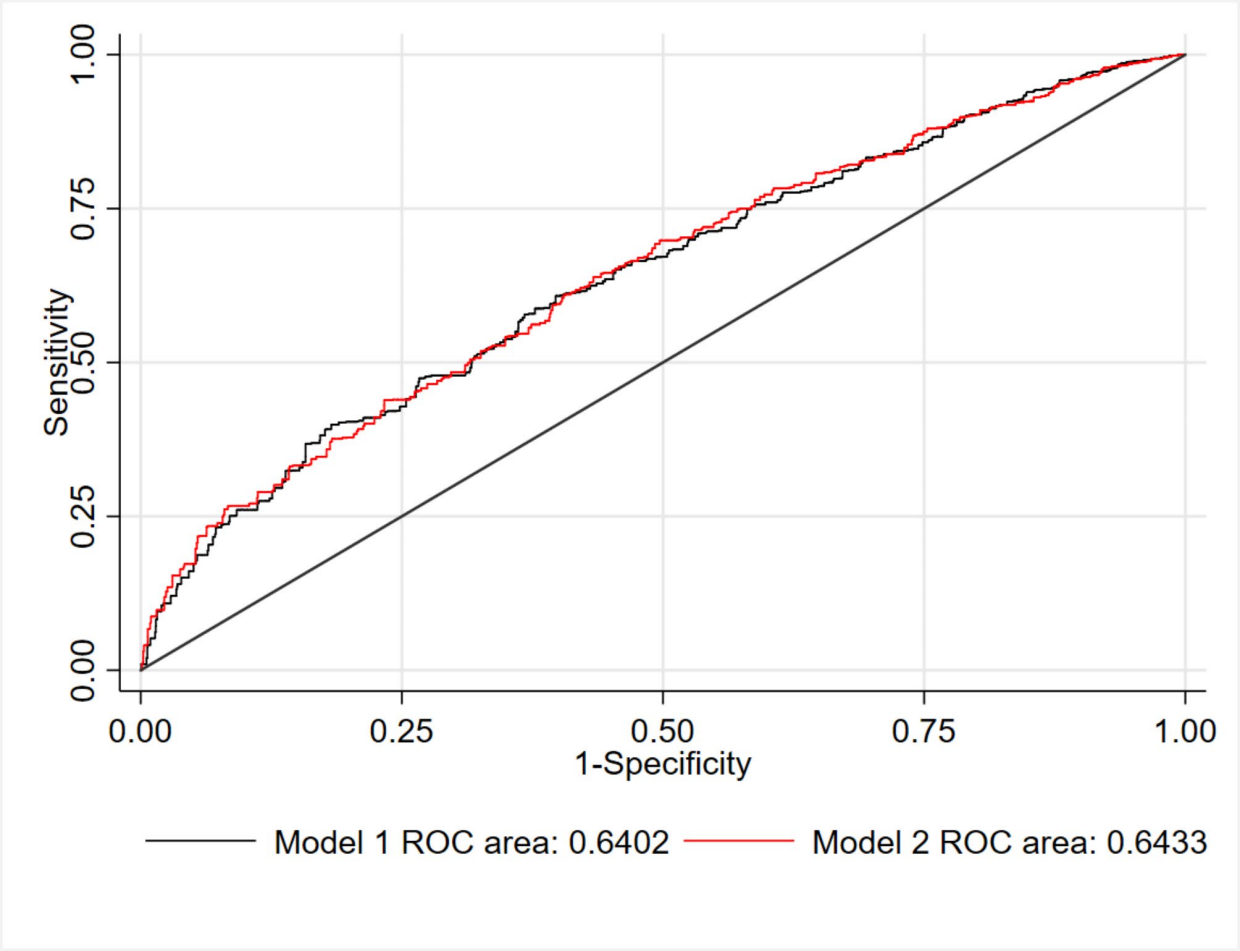
The area under the curve (AUC) of multivariable logistic model predicts high myopia. Model 1: age, sex, ethnicity, TV/computer usage, serum vitamin D level, and education attainment. Model 2: Model 1 plus serum vitamin A level

Every 1 µmol/L increment of serum vitamin A was associated with higher prevalence of high myopia (odd ratio = 1.46, 95%CI 1.01–2.10, P = 0.045) after adjusting for covariables described above. High levels of serum vitamin A was also negatively associated with the mean spherical equivalent of both eyes, before and after adjusted for covariables (Fig. 2). Furthermore, serum vitamin A concentration in the general population aged 12–25 ranged from 0.9 µmol/L (Percentile 1st ) to 3.0 µmol/L (Percentile 99st ). We did not detect a non-linear association between vitamin A and spherical equivalent among the general population (Supplementary Fig. 1). However, additional vitamin A did not significantly improve the accuracy of multivariable logistic prediction (Fig. 3).

### Asthma, C-reactive protein, and the risk of myopia

Previous studies have indicated that anaphylactic disease (allergic conjunctivitis, allergic rhinitis) and proinflammatory factors (C-reactive protein) increase myopia’s risk and severity of myopia [17–19]. In Random Forest algorithm, we also identified the moderate importance of C-reactive protein in predicting high myopia. After adjusting for age, sex, ethnicity, TV/computer usage, serum vitamin D level, serum vitamin A level, and education attainment, we found that neither C-reactive protein nor asthma was associated with myopia or high myopia (Supplementary Table 4).

## Discussion

Using Random Forest algorithm, this study found several anthropometric measurements, nutritional and serological factors associated with high myopia. Combined with multivariable logistic regress, high levels of serum vitamin A was seemingly associated with an increased prevalence of high myopia (adjusted odd ratio = 1.46 for 1 µmol/L increment). Furthermore, we exhibited that neither C-reactive protein nor asthma increased the risk and severity of myopia.

High myopia-related complications have become one of the major causes of vision loss. Extensive efforts have been paid to retard the progression of myopia [20]. The etiology of myopia progression involves a complex interaction between genetic, environmental, and social-economic factors [20, 21]. However, a few studies have thoroughly explored multi-levels (demographic, physical examination, nutritional and serological, immunological, and past medical history exposures) in their association with high myopia. Recently, Harb et al.; used the NHANES 2003–2008 to examine the possible role of nutrition in myopia. This study analyzed body height and body mass index, demographics, serum vitamin D and glucose/insulin levels, and caffeine intake. However, only serum insulin level was found to be associated with myopia [22]. Their results partially contradict other studies that found Lower serum vitamin D is associated with an increased risk of myopia [23]. However, it is still unclear to what extent this association is confounded by time outdoors [24]. Besides serum vitamin D, we firstly found high levels of serum vitamin A was seemingly associated with an increased prevalence of high myopia. However, this association should be interpreted with caution, because we applied multiple logistic regression. The multiple testing can increase type I error. Through the large sample size, a p value of 0.045 should be considered as borderline significance.

Vitamin A, or retinol, is not the primary bioactive mediator of its function. Its derivative all-trans retinoic acid (atRA) and 11-cis retinal are key gene transcription regulators [25]. Evidence from a genome-wide association study revealed retinoic acid metabolism genes contribute to refractive error susceptibility [26]. Serum vitamin A status is closely and positively related to tissue atRA concentration [27, 28]. Although it lacks direct evidence that arRA concentration in the retina and choroid is closed related to serum vitamin A, evidence from Obrochta et al. revealed that decreased dietary vitamin A significantly reduces testis atRA concentration [28]. The blood-testis barrier is similar to the blood-ocular barrier in selectively allowing serum components to enter the testes [29]. Thus, as the testis arRA is closely related to serum vitamin A, arRA concentration in the retina and choroid may also be closed related to serum vitamin A. Elevated atRA was found in both form-deprived and lens-induced myopia models in mammal animals [30–32]. Furthermore, feeding guinea pigs or chicks with atRA supplementations resulted in rapid eye elongation, companied by thinning of the retina and sclera [33, 34], which is similar to the pathophysiological change in myopia progression. Recently, Raine Study Gen2 found that adequate vitamin A at year 20 was associated with a borderline higher risk of myopia (OR, 1.57; 95% CI, 0.98–2.52; *P* = 0.06) [35].

The mechanism of atRA in promoting myopia progress involves extracellular matrix metabolism alteration. Scleral thinning, decreased scleral collagen I synthesis, glycosaminoglycan, and increased matrix metalloproteinase-2 have been documented in myopia development [36, 37]. Through the miR-328 -PAX6 axis, exogenous atRA could upregulate metalloproteinase-2 and downregulate collagen I and integrin *β*1 in the scleral cells [38]. In addition, atRA could stimulate retinal pigment epithelium to secret TGF-β, another strong extracellular matrix remodeling promoter [39].

In this present study, we found that neither C-reactive protein nor asthma increased the risk and severity of myopia, which partially contradicted previous studies. The anaphylactic disease was found to be linked with myopia. A large case-control combined prospective cohort study showed that allergic conjunctivitis is associated with a higher risk of myopia (hazard ratio = 2.35, 95% CI 2.29–2.40) after adjusting for age, gender, parent occupation, and residential area (urban/rural) [17]. The presence of allergic rhinitis (OR = 1.32, 95% CI 1.30–1.34) and atopic dermatitis (OR = 1.06, 95% CI 1.04–1.09) is also significantly associated with myopia [17]. Evidence also shows that allergy to indoor antigens (such as dust mites) is significantly related to myopia, while allergies to outdoor antigens are not statistically associated with myopia [40]. There is also evidence that outdoor antigens are significantly associated with high myopia (odds ratio = 2.67, 95% CI 1.57–4.51) [18]. The animal model revealed that administrating TNF-α and IL-6 to the conjunctival sac significantly promoted axial elongation and increased refractive error [17]. Together with the results of our study, it is reasonable to assume that local proinflammation factors, rather than systematic factors (such as C-reactive protein), promote myopia onset and progression. Yet, because allergic conjunctivitis and general allergic symptoms are associated with lesser time spent outdoors [41, 42], the observed association between local inflammation and myopia may be confounded by less time spent outdoors.

Our study has some strengths, including using the national cohort with Random Forest to select important multi-level exposures related to high myopia. However, limitations should be mentioned. First, we found high levels of serum vitamin A was associated with more significant refractive error and high myopia. But adding vitamin A concentration into the multivariable logistic regression did not significantly improve prediction accuracy (area under the curve from 0.640 to 0.643, P > 0.10). This result demonstrated that high myopia is a disease with multi-etiology involvement. Any attempt to predict high myopia from a single aspect, such as genetic, environmental, and social-economic factors, would not obtain desirable accuracy [21]. Second, the Raine study found that diet vitamin A intake during adolescence was not related to refractive errors at age 20, after adjustment for confounders [35]. However, no serum measurements of vitamin A levels were available in the Raine study. Thus, it still needs further investigation regarding the association between serum vitamin A and myopia severity. Third, we defined high myopia as the spherical equivalent of any eye ≤ − 5.00D. There might be a slight difference in the results of other articles with different definitions. Because high levels of serum vitamin A was also negatively associated with both eyes’ mean spherical equivalent, such different definitions would not lead to contradictory results. Fifth, refractive error was measured without cycloplegia, leading to overestimating myopia’s prevalence. In addition, a longitudinal study with group-based trajectory modeling is needed to explore the long-term effect of vitamin A on myopia onset and progression.

## Conclusion

High levels of serum vitamin A was seemingly associated with an increased prevalence of high myopia. This borderline significant association should be interpreted with caution because the potential increased type I error after the multiple testing. Neither C-reactive protein nor asthma was associated with an increased risk and severity of myopia, indicating local proinflammation factors rather than systematic factors could promote myopia onset and progression. In the future, multi-dimensional models combining genetic, environmental, and social-economic factors are needed to obtain desirable prediction accuracy in high myopia detection.

## Electronic supplementary material

Below is the link to the electronic supplementary material.

Supplementary Material 1

## Data Availability

The datasets generated and analyzed during the current study are available in the NHANES repository. https://wwwn.cdc.gov/Nchs/Nhanes/.
