# Supplementary material for: Screening for novel risk factors related to high myopia using machine learning
Source: BMC Ophthalmol. 2022 Oct 13;22:405. doi: 10.1186/s12886-022-02627-0 (PMC9558412; doi:10.1186/s12886-022-02627-0)
Supplement: Supplementary file 1 — Supplementary Material 1 [file 12886_2022_2627_MOESM1_ESM.docx]

**Supplementary Figure 1.** Scatter plot and restrictive cubic spline reveal linear association between serum vitamin A and mean spherical equivalent (D)

**
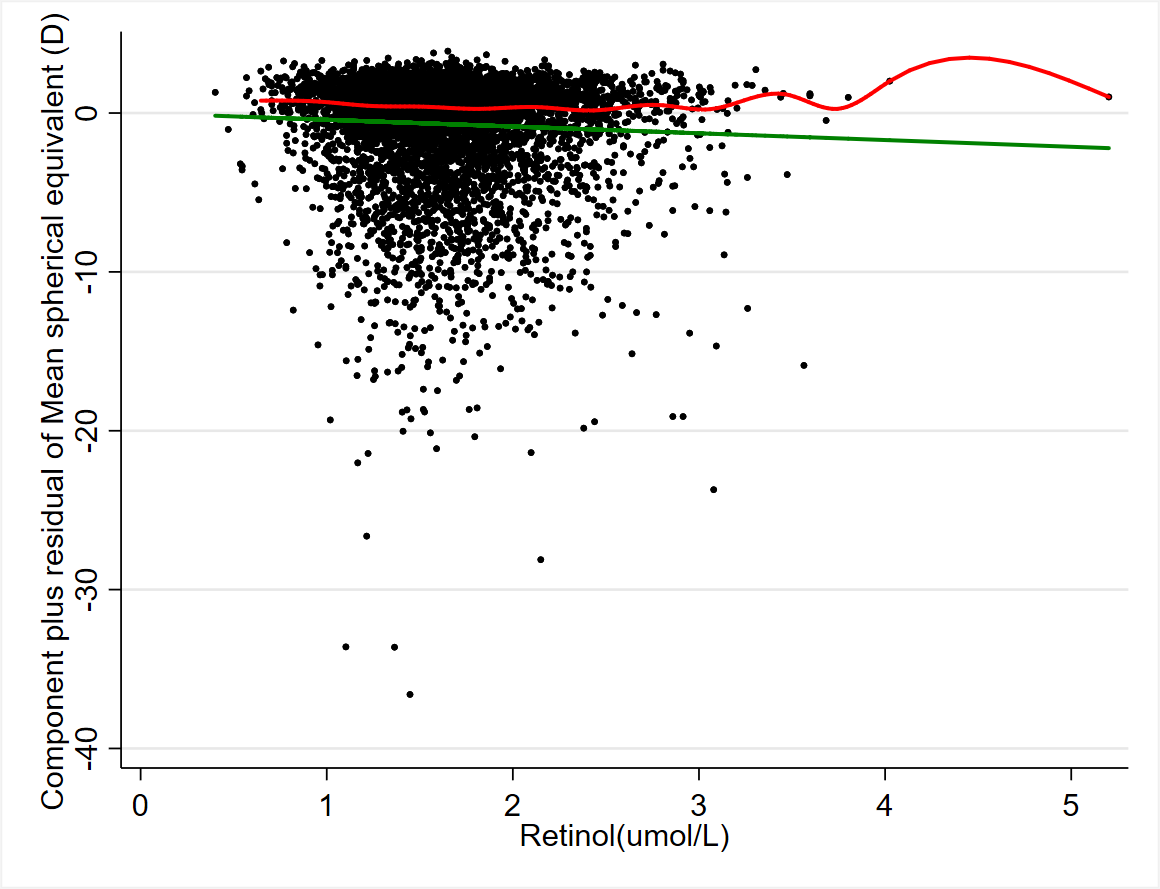
**

Green line: linear fit of scatter plot; Red line: restrictive cubic spline.

mean spherical equivalent is calculated by the mean of left and right spherical equivalent.

**Supplementary Table 1.** Included variable in this study.

| Variable label | Variable name |  |
| --- | --- | --- |
| **Demographic variables** | |  |
| Age (year) | ridageyr |  |
| Gender | riagendr |  |
| Education attainment | Derived variable | Categorical variable: <9th Grade, 9-11th Grade, High School Grade and Some College or above |
| Annual Household Income | indhhinc |  |
| **Physical examination variables** | |  |
| Systolic blood pressure (mmHg) | bpxsar |  |
| Diastolic blood pressure (mmHg) | bpxdar |  |
| BMI | bmxbmi |  |
| Waist Circumference (cm) | bmxwaist |  |
| Triceps Skinfold (mm) | bmxtri |  |
| Subscapular Skinfold (mm) | bmxsub |  |
| **Nutritional variables** | |  |
| Cadmium (ug/L) | lbxbcd |  |
| Lead (ug/dL) | lbxbpb |  |
| Folate, RBC (nmol/L RBC) | lbdrbfsi |  |
| Mercury, total (ug/L) | lbxthg |  |
| Homocysteine (umol/L) | lbxhcy |  |
| Ferritin (ng/mL) | lbxfer |  |
| Vitamin B12, serum (pg/mL) | lbxb12 |  |
| Vitamin D (nmol/L) | lbdvidms |  |
| Folate, serum (nmol/L) | lbdfolsi |  |
| Cotinine (ng/mL) | lbxcot |  |
| Total cholesterol (mmol/L) | lbdtcsi |  |
| HDL-cholesterol (mmol/L) | lbdhdlsi |  |
| HbA1c (%) | lbxgh |  |
| Total iron binding capacity (ug/dL) | lbxtib |  |
| Transferrin saturation (%) | lbdpct |  |
| Fast plasma glucose (mmol/L) | lbdglusi |  |
| Albumin (g/dl) | lbxsal |  |
| Alanine aminotransferase:SI (U/L) | lbxsatsi |  |
| Aspartate transaminase:SI(u/l) | lbxsassi |  |
| Alkaline phosphatase (U/L) | lbdsapsi |  |
| Blood urea nitrogen (mg/dL) | lbdsbusi |  |
| Total Calcium (mmol/L) | lbdscasi |  |
| Bicarbonate (mmol/L) | lbxsc3si |  |
| γ-glutamyl transpeptidase (U/L) | lbxsgtsi |  |
| Glucose, serum (mmol/L) | lbdsglsi |  |
| Iron (ug/dL) | lbxsir |  |
| Lactate dehydrogenase (U/L) | lbdsldsi |  |
| Phosphorus (mmol/L) | lbdsphsi |  |
| Bilirubin, total (umol/L) | lbdstbsi |  |
| Total protein (g/dL) | lbxstp |  |
| Triglycerides (mmol/L) | lbdstrsi |  |
| Uric acid (umol/L) | lbdsuasi |  |
| Creatinine (mg/dL) | lbdscr |  |
| Sodium (mmol/L) | lbxsnasi |  |
| Potassium (mmol/L) | lbxsksi |  |
| Chloride (mmol/L) | lbxsclsi |  |
| Osmolality (mmol/Kg) | lbxsossi |  |
| Globulin (g/dL) | lbxsgb |  |
| Vitamin E(ug/dl) | lbxvie |  |
| Vitamin A(ug/dl) | lbdviasi |  |
| total b-carotene (ug/dl) | lbxbcc |  |
| Vitamin B6 (pg/L) | lbxvb6 |  |
| eGFR (mL/min) | Derived variable | Continuous variable: creatinine-only CKD-EPI formula |
| **Blood test variables** | |  |
| Hemoglobin (g/dL) | lbxhgb |  |
| CRP (mg/dl) | lbxcrp |  |
| **Immunological Variable** | |  |
| CMV antibody | sscmv |  |
| Hepatitis A antibody | lbxha |  |
| Hepatitis B surface antibody | lbxhbs |  |
| Hepatitis B core antibody | lbxhbc |  |
| Hepatitis C antibody | lbdhcv |  |
| Measles antibody | lbxme |  |
| Rubella international units | lbdruiu |  |
| Varicella antibody | lbxvar |  |
| Toxoplasma antibody (IgG) | lbdto1 |  |
|  |  |  |
| **Past medical history variables** | |  |
| Diabetes | Derived variable | Continuous variable: fasting plasma glucose ≥ 126 mg/dL (7.0 mmol/L) or 2-hour plasma glucose after OGTT test ≥ 200 mg/dL (11.1 mmol/L) or A1C ≥ 6.5%. |
| Prediabetes | Derived variable | Continuous variable: 100 mg/dL (5.6 mmol/L) ≤ fasting plasma glucose ≤ 125 mg/dL (6.9 mmol/L), or 140 mg/dL (7.8 mmol/L) ≤ 2-hour plasma glucose after OGTT test ≤ 199 mg/dL (11.0 mmol/L), or 5.7% ≤ HbA1c ≤ 6.4% |
| Asthma | mcq010 |  |
| Age when first had asthma | mcq025 |  |
| Asthma duration | Derived variable | Continuous variable: ridageyr-mcq025 |
| Still have asthma | mcq035 |  |
| MET score | Derived variable | Categorical variable: Detail in main text |
| TV and computer use | Derived variable | Continuous variable: PAD480 for NHANES2001-2002; PAD590+PAD600 for NHANES2003-2008 |
| Smoker | Derived variable | Categorical variable: smoker as a participant who smoked every day (smq20) |
| Household smoker | smd410 |  |

**Supplementary Table 2.** Cross-sectional comparison of included participant.

|  | No myopia (n=3205) | Mild and moderate myopia (n=3459) | High myopia (n=368) | P-value |
| --- | --- | --- | --- | --- |
| Annual Household Income % |  |  |  | 0.37 |
| $ 0 to $ 4,999 | 2.2 (0.4) | 2.3 (0.5) | 5.1 (2.2) |  |
| $ 5,000 to $ 9,999 | 4.3 (0.6) | 4.0 (0.7) | 3.6 (1.3) |  |
| $10,000 to $14,999 | 6.7 (0.6) | 6.5 (0.7) | 4.5 (1.4) |  |
| $15,000 to $19,999 | 8.5 (1.4) | 6.4 (0.7) | 3.8 (1.3) |  |
| $20,000 to $24,999 | 6.5 (0.7) | 6.5 (0.7) | 8.0 (1.9) |  |
| $25,000 to $34,999 | 11.8 (1.2) | 11.8 (1.0) | 12.9 (2.4) |  |
| $35,000 to $44,999 | 9.2 (0.7) | 9.6 (0.8) | 9.8 (2.2) |  |
| $45,000 to $54,999 | 8.5 (0.7) | 9.0 (1.0) | 12.9 (3.1) |  |
| $55,000 to $64,999 | 7.7 (0.8) | 8.4 (0.9) | 6.3 (1.8) |  |
| $65,000 to $74,999 | 6.1 (0.8) | 7.3 (0.9) | 7.8 (2.0) |  |
| $75,000 and over | 28.4 (1.7) | 28.2 (1.5) | 25.3 (3.4) |  |
| Systolic blood pressure (mmHg) | 110.8 (0.4) | 111.1 (0.4) | 109.9 (0.9) | 0.84 |
| Diastolic blood pressure (mmHg) | 61.8 (0.4) | 62.6 (0.4) | 63.8 (0.8) | 0.017 |
| BMI | 24.7 (0.2) | 24.9 (0.2) | 24.5 (0.4) | 0.74 |
| Waist Circumference (cm) | 84.9 (0.5) | 85.5 (0.5) | 84.6 (1.1) | 0.69 |
| Triceps Skinfold (mm) | 16.5 (0.2) | 16.9 (0.2) | 18.6 (0.5) | 0.001 |
| Subscapular Skinfold (mm) | 14.7 (0.2) | 15.0 (0.2) | 15.6 (0.4) | 0.024 |
| Cadmium (ug/L) | 0.20 [0.14-0.40] | 0.20 [0.14-0.40] | 0.20 [0.14-0.31] | 0.011 |
| Lead (ug/dL) | 0.90 [0.65-1.32] | 0.90 [0.60-1.29] | 0.82 [0.60-1.10] | <0.001 |
| Folate, RBC (nmol/L RBC) | 521 [428-639] | 534 [439-650] | 530 ]451-652] | 0.246 |
| Mercury, total (ug/L) | 0.53 [0.23-1.10] | 0.55 [0.30-1.17] | 0.64 [0.30-1.60] | 0.12 |
| Homocysteine (umol/L) | 6.47 [5.42-7.82] | 6.60 [5.47-7.94] | 6.54 [5.65-7.76] | 0.46 |
| Ferritin (ng/mL) | 35 [21-61] | 35 [20-61] | 34 [21-59] | 0.9 |
| Vitamin B12, serum (pg/mL) | 485 [371-613] | 478 [372-624] | 477 [335-594] | 0.17 |
| Vitamin D (nmol/L) | 65.8 [51.7-80.0] | 63.7 [51.7-78.2] | 62.3 [46.8-78.2] | 0.058 |
| Folate, serum (nmol/L) | 25.1 [19.0-33.3] | 25.1 [18.8-33.5] | 26.0 [20.2-32.2] | 0.63 |
| Cotinine (ng/mL) | 0.20 [0.03-14.6] | 0.14 [0.03-4.30] | 0.08 [0.02-1.30] | 0.004 |
| Total cholesterol (mmol/L) | 4.35 (0.03) | 4.33 (0.03) | 4.37 (0.08) | 0.78 |
| HDL-cholesterol (mmol/L) | 1.32 (0.01) | 1.34 (0.01) | 1.38 (0.03) | 0.032 |
| HbA1c (%) | 5.11 (0.01) | 5.13 (0.02) | 5.12 (0.03) | 0.32 |
| Total iron binding capacity (ug/dL) | 377 (2) | 377 (2) | 380 (7) | 0.72 |
| Transferrin saturation (%) | 24.7 (0.5) | 24.2 (0.5) | 22.8 (1.1) | 0.19 |
| Fast plasma glucose (mmol/L) | 5.09 (0.03) | 5.15 (0.04) | 5.24 (0.10) | 0.14 |
| Albumin (g/dl) | 4.38 (0.01) | 4.38 (0.01) | 4.40 (0.02) | 0.78 |
| Alanine aminotransferase:SI (U/L) | 17 [14-23] | 18 [14-24] | 17 [14-23] | >0.9 |
| Aspartate transaminase:SI(u/l) | 22 [19-26] | 22 [19-26] | 21 [18-24] | 0.36 |
| Alkaline phosphatase (U/L) | 79 [61-122] | 77 [59-120] | 69 [55-90] | 0.002 |
| Blood urea nitrogen (mg/dL) | 3.57 [2.86-4.64] | 3.57 [2.86-4.64] | 3.57 [2.86-4.28] | 0.71 |
| Total Calcium (mmol/L) | 2.411 (0.003) | 2.416 (0.003) | 2.409 (0.007) | 0.44 |
| Bicarbonate (mmol/L) | 24.0 (0.1) | 24.0 (0.1) | 24.1 (0.1) | 0.6 |
| γ-glutamyl transpeptidase (U/L) | 14 [11-19] | 14 [11-19] | 14 [10-19] | 0.54 |
| Glucose, serum (mmol/L) | 4.78 (0.02) | 4.81 (0.03) | 4.83 (0.06) | 0.25 |
| Iron (ug/dL) | 87 [64-116] | 86 [62-115] | 82 [58-113] | 0.12 |
| Lactate dehydrogenase (U/L) | 123 [109-142] | 122 [108-141] | 116 [104-113] | 0.003 |
| Phosphorus (mmol/L) | 1.36 [1.23-1.49] | 1.32 [1.20-1.48] | 1.29 [1.20-1.45] | 0.22 |
| Bilirubin, total (umol/L) | 12.0 [10.3-15.4] | 12.0 [10.3-15.4] | 12.0 [10.3-15.4] | 0.52 |
| Total protein (g/dL) | 7.29 (0.02) | 7.30 (0.02) | 7.30 (0.04) | 0.71 |
| Triglycerides (mmol/L) | 1.15 (0.03) | 1.16 (0.03) | 1.14 (0.06) | 0.92 |
| Uric acid (umol/L) | 303 [250-357] | 297 [250-357] | 297 [244-351] | 0.4 |
| Creatinine (umol/L) | 70.7 [61.9-79.6] | 70.7 [61.9-79.6] | 70.7 [61.9-79.6] | 0.48 |
| Sodium (mmol/L) | 139 [138-140] | 139 [138-140] | 139 [138-140] | 0.07 |
| Potassium (mmol/L) | 4.0 [3.8-4.2] | 4.0 [3.8-4.2] | 3.9 [3.7-4.1] | 0.33 |
| Chloride (mmol/L) | 104 [102-105] | 104 [102-105] | 104 [102-105] | 0.65 |
| Osmolality (mmol/Kg) | 276 [273-278] | 276 [274-279] | 276 [274-279] | 0.14 |
| Globulin (g/dL) | 2.9 [2.6-3.2] | 2.9 [2.7-3.2] | 2.9 [2.7-3.1] | 0.79 |
| Vitamin E(ug/dl) | 882 [733-1078] | 876 [733-1059] | 937 [783-1146] | 0.16 |
| Vitamin A(ug/dl) | 49.3 [41.6-58.8] | 49.1 [41.0-58.1] | 50.6 [42.4-61.3] | 0.52 |
| total b-carotene (ug/dl) | 9.4 [6.3-15.1] | 6.2 [6.2-14.4] | 9.7 [6.4-20.8] | 0.076 |
| Vitamin B6 (pg/L) | 485 [371-613] | 478 [372-624] | 477 [335-594] | 0.065 |
| eGFR (mL/min) | 119 (0.9) | 120 (0.9) | 121 (1.6) | 0.32 |
| Hemoglobin (g/dL) | 14.5 (0.1) | 14.5 (0.1) | 14.3 (0.1) | 0.041 |
| CRP (mg/dl) | 0.06 [0.02-0.20] | 0.07 [0.03-0.24] | 0.08 [0.03-0.30] | 0.076 |
| CMV antibody (% positive) | 47.4 (2.6) | 43.5 (2.2) | 36.0 (4.0) | 0.024 |
| Hepatitis A antibody (% positive) | 26.3 (2.1) | 25.0 (1.6) | 23.2 (4.1) | 0.53 |
| Hepatitis B surface antibody  (% positive) | 50.9 (2.9) | 50.7 (1.4) | 51.4 (3.6) | >0.90 |
| Hepatitis B core antibody (% positive) | 1.3 (0.3) | 1.6 (0.4) | 2.1 (1.2) | 0.65 |
| Hepatitis C antibody (% positive) | 0.009 (0.009) | 0.05 (0.04) | 0 | 0.46 |
| Measles antibody (units) | 7.0 [3.7-11.8] | 6.9 [3.5-11.6] | 6.0 [3.1-11.2] | 0.46 |
| Rubella international units | 40 [21-66] | 36 [20-65] | 31 [20-56] | 0.14 |
| Varicella antibody | 13.1 [8.1-18.6] | 13.2 [7.6-18.4] | 14.6 [8.4-18.8] | 0.88 |
| Toxoplasma antibody (IgG) | 0 [0-91] | 0 [0-53] | 0 [0-13] | 0.10 |
| Diabetes (%) | 0.49 (0.15) | 0.95 (0.24) | 1.25 (0.82) | 0.26 |
| Prediabetes (%) | 7.1 (0.6) | 7.1 (0.7) | 6.5 (1.8) | 0.93 |
| Asthma (%) | 18.8 (1.2) | 17.4 (0.9) | 11.5 (1.8) | 0.02 |
| Age when first had asthma | 7 [3-12] | 8 [4-12] | 8 [4-14] | 0.56 |
| Asthma duration (years) | 11 [6-15] | 10 [6-14] | 8 [5-15] | 0.3 |
| Still have asthma  (% among asthma participants) | 46.5 (3.5) | 43.3 (3.1) | 38.2 (8.8) | 0.64 |
| MET score | 748 [21-980] | 741 [22-957] | 752 [672-910] | 0.38 |
| TV and computer use (hours/day) | 3 [1-4] | 3 [2-5] | 3 [1-5] | 0.2 |
| Smoker (%) | 26.3 (1.8) | 24.5 (1.1) | 22.3 (3.1) | 0.33 |
| Household smoker (%) | 23.3 (1.9) | 21.4 (13.3) | 17.0 (3.0) | 0.14 |

Data were weighted estimates, and expressed as mean (standard error) or median [percentile 25 -percentile 75] when appropriate

**Supplementary Table 3.** Multi-variable logistic regression results of the association between Top 15 factors and high myopia.

|  | Myopia | | High Myopia | |
| --- | --- | --- | --- | --- |
|  | OR | 95% CI | OR | 95% CI |
| Hemoglobin | 0.99 | 0.92-1.07 | 0.92 | 0.77-1.10 |
| eGFR | 1.00 | 0.99-1.00 | 1.00 | 0.99-1.01 |
| Serum Phosphorus | 1.01 | 0.70-1.46 | 1.24 | 0.55-2.79 |
| Vitamin A | 1.01 | 0.83-1.24 | **1.46** | **1.01-2.10** |
| Transferrin Saturation | 1.00 | 0.99-1.00 | 0.99 | 0.97-1.00 |
| Alkaline Phosphatase | 1.00 | 1.00-1.00 | 1.00 | 0.99-1.00 |
| Albumin | 1.15 | 0.94-1.40 | **1.77** | **1.15-2.72** |
| Total cholesterol | 0.95 | 0.86-1.04 | 0.97 | 0.80-1.17 |
| Uric Acid | 1.00 | 1.00-1.00 | 1.00 | 1.00-1.00 |
| Total calcium | **4.02** | **1.44-11.22** | 4.20 | 0.52-33.68 |
| Iron | 1.00 | 1.00-1.00 | 1.00 | 0.99-1.00 |
| Homocysteine | 1.02 | 0.98-1.07 | 0.98 | 0.91-1.06 |
| Triglycerides | 1.00 | 0.90-1.10 | 0.99 | 0.83-1.17 |
| Log transformed C-reactive protein | 1.04 | 0.99-1.09 | 0.98 | 0.88-1.09 |

Data were weighted estimates. OR, odds ratio; 95% CI, 95% confidence interval.

**Supplementary Table 4.** Relationship between asthma, C-reactive protein with myopia.

|  | Myopia | | | High Myopia | | |
| --- | --- | --- | --- | --- | --- | --- |
|  | aOR | 95% CI | P-value | aOR | 95% CI | P-value |
| Log-transformed C-reactive protein (per 1-unit increment) | 1.03 | 0.98-1.09 | 0.20 | 0.97 | 0.87-1.09 | 0.63 |
| Still have asthma (Yes vs. No) | 1.00 | 0.73-1.35 | >0.9 | 0.73 | 0.41-1.29 | 0.27 |
| Asthma duration  (per 1-year increment) | 1.00 | 0.96-1.03 | 0.79 | 0.98 | 0.89-1.09 | 0.70 |
| Asthma attack in past year  (Yes vs. No) | 0.76 | 0.52-1.11 | 0.15 | 0.82 | 0.44-1.54 | 0.54 |

Data were weighted estimates. aOR, adjusted odds ratio; 95% CI, 95% confidence interval. Multivariable logistic regression adjusted for age, sex, ethnicity, TV/computer usage, serum vitamin D level, serum vitamin A level and education attainment
